# Expression of OCT4A: The First Step to the Next Stage of Urothelial Bladder Cancer Progression

**DOI:** 10.3390/ijms150916069

**Published:** 2014-09-11

**Authors:** Wojciech Jóźwicki, Anna A. Brożyna, Jerzy Siekiera

**Affiliations:** 1 Department of Tumor Pathology and Pathomorphology, the Ludwik Rydygier Collegium Medicum in Bydgoszcz, Nicolaus Copernicus University in Torun, Romanowska Street 2, Bydgoszcz 85-796, Poland; E-Mail: anna.brozyna@cm.umk.pl; 2 Department of Tumor Pathology and Pathomorphology, the Franciszek Łukaszczyk Oncology Centre, Romanowska Street 2, Bydgoszcz 85-796, Poland; 3 Department of Urology, the Franciszek Łukaszczyk Oncology Centre, Romanowska Street 2, Bydgoszcz 85-796, Poland, E-Mail: siekieraj@co.bydgoszcz.pl

**Keywords:** OCT4, urothelial bladder cancer, progression, prognosis, cancer stem cells

## Abstract

OCT4 (octamer-binding transcription factor) is a transcription factor responsible for maintaining the pluripotent properties of embryonic stem cells. In this paper, we present the results of studies to investigate the role of the OCT4 splicing variant in urothelial bladder cancer and the relationship between the OCT4 phenotype and the morphological parameters of tumor malignancy. Ninety patients who received a cystectomy for bladder cancer were enrolled. The expression of OCT4 protein was analyzed by immunohistochemistry. The ratio of OCT4-positive cells was the lowest in pT1 (pathological assessment (p)—tumor extent confined to mucosa (T1)) tumors and the highest in pTis (non-papillary tumor extent confined to urothelium) and pT2 (tumor extent including muscularis propria) tumors. Information about the percentage of OCT4A-positive tumor cells could facilitate choosing the treatment mode in borderline pTis–pT1 (crossing the border of the basement membrane; the first stage of progression) and pT1–pT2 (crossing the border of the muscularis propria; the second stage of progression) cases: a higher percentage of OCT4A-positive cells should support more radical therapy. A significantly higher percentage of cases with moderate OCT4 intensity was found in metastasizing (the third stage of progression) cases with >2 positive lymph nodes. The percentage of OCT4-positive cells was significantly higher for cancers with a high grade, higher non-classic differentiation number and greater aggressiveness of invasion. The differentiation, maturation and aggressiveness of tumor invasion appear to depend on the expression of the OCT4 phenotype in cancer cells, similar to the successive stages of malignancy progression in urothelial cancer.

## 1. Introduction

Urothelial cancers are cancers of relatively unusual biology. One group of urothelial cancers includes non-invasive cancers, such as papillary and* in situ* cancer, and another group includes invasive cancers that infiltrate the mucosa or muscularis propria (“non-muscle invasive” and “muscle invasive”, respectively) [1]. Biological differences within each category are significant. The so-called surface cancers (pTa, pT1) (papillary tumor extent confined to urothelium (pTa), tumor extent confined to mucosa (pT1)) tend to exhibit a weak invasive tendency, in contrast to cancers that infiltrate the deep layers (pT2–4: tumor extent including muscularis propria (pT2), perivesical fat (pT3) or perivesical organs (pT4)), which show a strong tendency for multidirectional differentiation (non-classic differentiation number, NDN) and a high metastatic potential [1,2,3]. Although* in situ* cancers are non-invasive, they have a high invasive potential and, in cases that progress, reach an advanced stage quickly [4,5].

The diverse types of biological malignancy of urothelial bladder cancers forms the basis of the different clinical management standards, which include: (1) conservative management for papillary (pTa) and surface-infiltrating (pT1) cancers; (2) radical treatment for cancers infiltrating the deep layers (pT2–4); and (3) conservative and radical treatment for* in situ* cancers (pTis). Application of these treatment standards is clear for cases with well-defined progression. However, decisions are not as clear for so-called borderline cases, in which the potential therapeutic benefits for a patient are difficult to assess unambiguously, and raise the following questions: Does the presence of numerous* in situ* cancer foci justify a radical procedure? Do more frequent recurrences of surface high-grade pT1 cancer justify a radical procedure? Is it necessary to introduce a more radical procedure for a tumor at the borderline pT1–pT2 stage with suspected focal invasion to the muscularis propria in a patient in a very good clinical condition, or can we wait a little longer and give the patient more time before the bladder must be removed? From a statistical point of view, the answers seem clear, but they are less clear when applied to a specific individual patient. Studies have aimed to find markers of progression and prognosis that would facilitate individualization of therapeutic decisions. Attempts have been made to study the usefulness of various cell cycle- and proliferation-related biomarkers that may be diagnostically useful [6,7]. Researchers have also focused on stem cells [8] and certain aspects of their biology in the process of carcinogenesis; e.g., origination of cancer stem cells [9].

OCT4 (OCT3, octamer-binding transcription factor, also known as POU5F1) is a transcription factor that participates in the maintenance of the pluripotent properties of embryonic stem cells. In a mature organism, OCT4A is not present in mature and differentiated cells and is found only in germ cells [10,11]. OCT4 protein is encoded by the octamer-binding transcription factor 4 (*OCT4*) gene, although its isoforms can be created by alterative splicing and alternative translation [12,13,14]. The functions of individual isoforms have not been studied in detail and are not understood fully. Only the OCT4 form, which is present in cell nuclei, exhibits transcription factor functions and is responsible for maintaining cells at an undifferentiated stage, stem cell properties and ability for self-renewal. OCT4A, as a transcription factor, regulates the expression of several target genes, including *NANOG*, *SOX-2*, *REX-1* and *CDX-2*, involved in the regulation of pluripotency. OCT4 is generally considered a universal marker of pluripotent stem cells [13].

OCT4 is expressed in cancer cells [15,16,17,18]. The presence of OCT4 protein is associated with, e.g., poor prognosis in non-small-cell lung cancer [15], hepatic cancer [17] and esophageal squamous cell carcinoma [19]. One possible mechanism responsible for the more aggressive behavior of cancers and worse clinical outcomes with cells expressing OCT4 is the presence of the stem cell phenotype in cancers related to OCT4-mediated dedifferentiation [20] and related chemoresistance [21,22,23]. Some reports have suggested that OCT4 protein may be present in neoplastic lesions in the urinary bladder [24,25]. However, the authors of these studies did not analyze splicing or translation variants, but only the total OCT4 protein level in bladder cancer cells.

In this paper, we present the results of our studies to investigate the expression level of the OCT4 splicing variant as a stem cell marker in samples of urothelial bladder cancer obtained from primary tumors and metastases to regional lymph nodes. We used immunohistochemistry to study the relationships between the intensity of OCT4 expression and the percentage of OCT4-positive cells in the tumor and the histological phenotype determined by the progression stage (pT, staging), histological differentiation (G, grading), tissue invasion type (TIT) and NDN [1,2,3,26]. We also analyzed these results in relation to the subsequent steps of tumor progression, defined as follows: the first stage of progression when a non-invasive* in situ* lesion progresses to invasion, the second stage when bladder cancer cells invade the muscle layer (muscularis propria) in the bladder wall and the third stage when bladder cancers start to metastasize.

## 2. Results

Consistent with the characteristics of the OCT4A variant and with our previous research [27], only nuclear immunostaining was observed. In all lesions examined, only low or moderate staining intensity was seen. Within the primary tumor and metastases to lymph nodes, OCT4A-positive cells represented, on average, 3.47% of all cancer cells.

### 2.1. OCT4A and Staging

We observed a significant variation in the percentage of OCT4A-positive cells in individual cases (from 0%–80%). The detailed percentages of the cases classified with low and/or moderate intensity of OCT4A expression are shown in Table 1.

The characteristics of patients with extreme percentages of OCT4A-positive cells are presented in Table 2.

**Table 1:** Percentages of cases with OCT4A expression within the urothelium.

Urothelial Tissue	Percentage of Cases			
	Low	Moderate	All	
			%	*p*
Non-neoplastic (normal urothelium)	25	0	25	>0.05
Primary tumor	81.7	18.1	84.3	>0.05
Primary metastasizing	82.6	17.4	91.3	>0.05
Primary non-metastasizing	80	20	81.7	>0.05
Lymph node metastasis	87	10	87	>0.05

**Table 2:** Characteristics of patients without OCT4A-positive cells and with 80% of OCT4A-positive cells. pT, pathological assessment (p) classifying the extent of cancer spread (T) by WHO TNM Classification of Malignant Tumours; pN, pathologic lymph node status; G, grading; NDN, non-classic differentiation number; TIT, tissue invasion type; n, number of cases.

Feature	Zero Per cent of OCT4A-Positive Cells (*n* = 16)	Eighty Per cent of OCT4A-Positive Cells (*n* = 3)
pT	a (*n* = 6), is (*n* = 1), 1 (*n* = 1), 2 (*n* = 2), 3 (*n* = 6)	is, 3, 4
pN	1 (*n* = 1), 2 (*n* = 1)	1
G	High (*n* = 10), low (*n* = 6)	High
NDN	0 (*n* = 10), 1 (*n* = 3), 2 (*n* = 2), 3 (*n* = 1)	2
TIT	Focal (*n* = 7), styloid (*n* = 1), dispersive (*n* = 1)	Focal, dispersive

**Figure 1:**
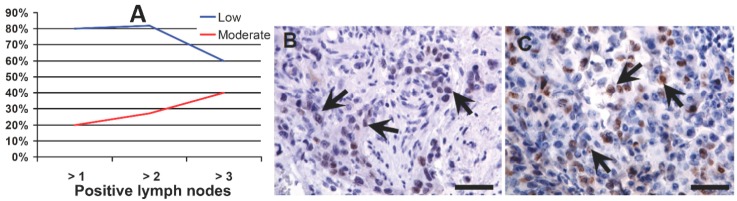
OCT4A expression in a primary tumor: percentage of cases (**A**) with low (**B**) and with moderate (**C**) expression in tumors with an increasing number of metastases to regional lymph nodes. Arrows show Oct4A-positive nuclei (visualized with diaminobenzidine (DAB) (brownish) and counterstained with hematoxylin (blue)); scale bars: 100 µm.

In each cancer category, the percentages of cases with low and/or moderate intensity staining of OCT4A were similar. However, more detailed analysis of metastatic tumors (progression to the third stage) showed that, in cases with >2 lymph nodes involved, the percentage of cases with moderate OCT4A staining intensity was significantly higher (Figure 1A–C).

**Figure 2:**
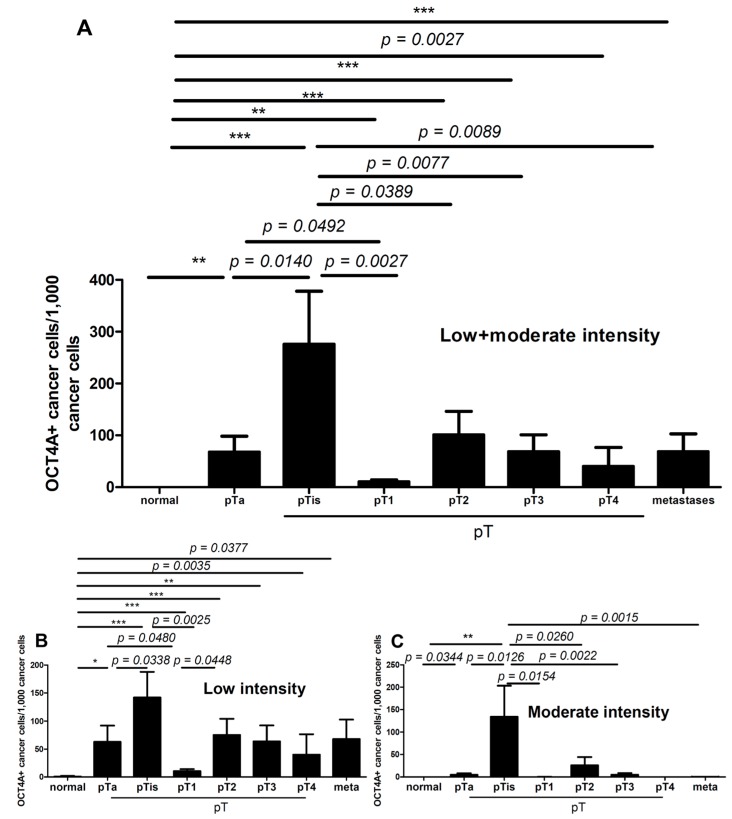
The mean (**A**) percentage of cells with low and moderate level of OCT4A in normal epithelium, primary tumors and metastases; The low (**B**) and moderate (**C**) mean expression of OCT4A in normal epithelium, primary tumors and metastases. Significant differences are denoted by *p*-values (*t*-test) and as * *p* < 0.05, ** *p* < 0.01, *** *p* < 0.001 by ANOVA.

The mean percentages of OCT4A-positive cells were high in non-invasive cancers (*in situ* and pTa) and in cancers infiltrating the muscularis propria (pT2–4). The lowest percentage of OCT4A-positive cells was found in pT1 tumors (Figure 2A). Similar results were found when the percentage of cells with low OCT4A staining intensity was analyzed (Figure 2B).

Analysis of the number of cells with moderate OCT4A staining intensity showed that they were the most numerous in pTis (the first step of the first stage of progression) and pT2 (first step of the second stage of progression) cancers and were significantly more abundant in these cancers compared with other cancer progression stages (pTis* vs.* pTa/pT1, Figure 2C; pT2* vs.* pT3–4 and metastases, *p* = 0.0082, data not shown).

**Figure 3:**
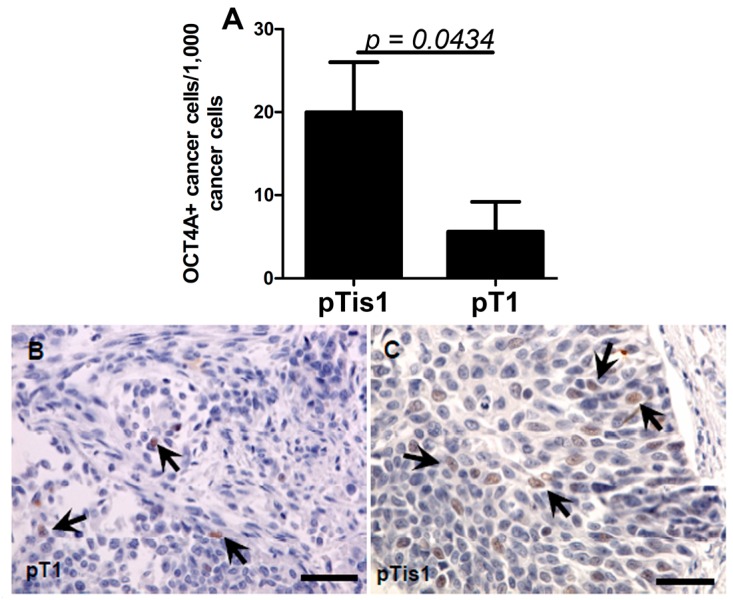
Mean low OCT4A level (**A**) in non-muscle invasive cancers without a coexisting* in situ* component (**B**) and with that component (**C**). Significant differences are denoted by *p*-values (*t*-test). Arrows show OCT4A-positive nuclei (visualized with DAB and counterstained with hematoxylin); scale bars: 50 μm.

The percentage of low-intensity OCT4A-positive cells was significantly lower in pT1 tumors compared with pTis1 (Figure 3A). Representative images of the immunostaining of pT1 and pTis1 tumors are shown in Figure 3B,C.

### 2.2. OCT4A and Grading

**Figure 4:**
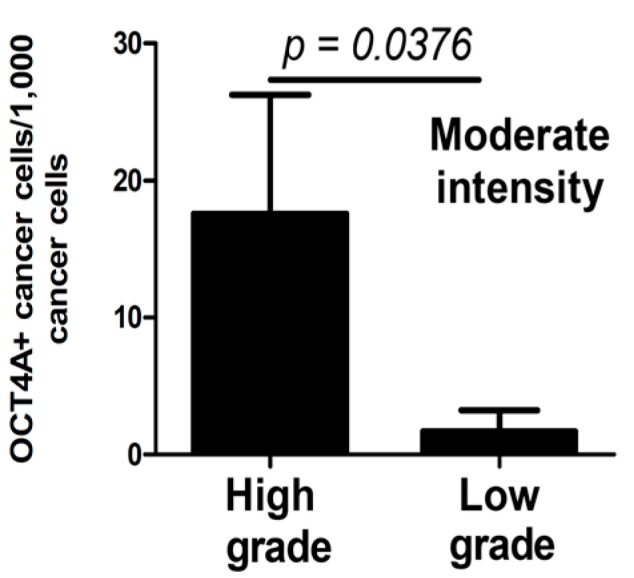
Percentage of OCT4A-positive cells showing moderate staining intensity in high- and low-grade tumors. Significant difference is denoted by *p*-values (*t*-test).

Moderate-intensity OCT4A expression was observed for a significantly higher percentage of cells in high-grade compared with low-grade tumors (*t*-test, *p* = 0.0364, Figure 4).

### 2.3. OCT4A and Tissue Invasion Type

**Figure 5:**
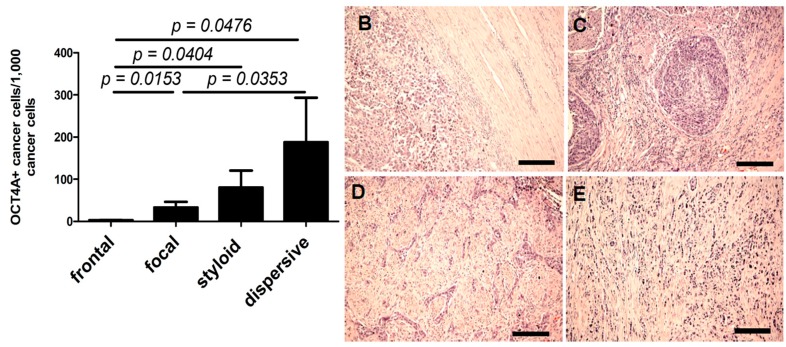
The percentage of low-intensity OCT4A-positive cells (**A**) was higher in tumors with higher aggressiveness of local invasion, styloid (**D**) and dispersive (**E**), compared with tumors with lower aggressiveness of local invasion, frontal (**B**) and focal (**C**). Scale bars: 100 µm. Significant differences are denoted by *p*-values (*t*-test).

A significant positive correlation was observed between the percentage of cells with low OCT4A expression and the aggressiveness of tumor invasion (*r* = 0.02994, *p* < 0.0001, Figure 5A–E). The lowest and the highest percentages of OCT4A-positive cells were observed in tumors with the lowest (Figure 5B) and the highest (Figure 5E) invasive potential, respectively.

### 2.4. OCT4A and NDN

**Figure 6:**
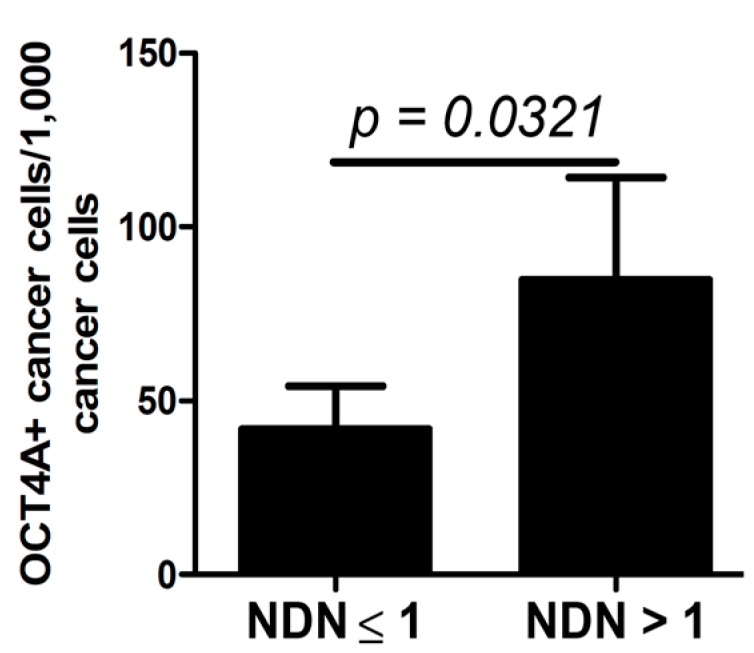
Percentage of low-intensity OCT4A-postive cells in relation to NDN status. The significant differences are denoted by *p*-values (*t*-test).

The percentage of low-intensity OCT4A-positive cells was significantly higher in tumors in which the NDN was >1 (Figure 6).

## 3. Discussion

Cancer stem cells represent a small subpopulation of cells that can initiate cancer and cause its recurrence and metastasis. These cells were isolated for the first time from a patient with acute myeloblastic leukemia [28,29]. In subsequent years, research results confirmed the presence of cells that expressed stem cell markers in other cancers [30], including brain, breast, prostate and lung tumors [31,32,33,34,35]. Cancer stem cells are relatively resistant to chemo- and radio-therapy [36,37,38], and possibly for this reason, the prognosis is poor for patients whose cancer expresses stem cell markers. Complete healing of the neoplastic process may be related to the elimination of all stem cells [38].

Few reports have suggested that OCT4A is expressed in urinary bladder lesions. However, results obtained with the use of antibodies to identify OCT4A isoforms, both A and B, do not fully explain the role of stem cells in bladder cancer. One of the first reports was by Atlasi* et al.* [24], who reported *OCT4A* expression in up to 96% of the cancers analyzed by reverse transcription-polymerase chain reaction (PCR). In contrast to our results, the percentage of OCT4A-positive cells was high, but there was no correlation between the OCT4A expression level and the clinical and histological tumor phenotype (stage, grade or size). These differences between studies may reflect the fact that Atlasi* et al.* [24] studied the expression level of OCT4A protein without distinguishing its isoforms, which may have significantly influenced the results obtained. Huang* et al.* [25] found that OCT4A expression was of prognostic importance in bladder cancers. In the group of patients with pTa or pT1 cancers, OCT4A expression was found more often in those with high-grade* vs.* low-grade cancers (75% of patients* vs.* 18% of patients, respectively), and its expression correlated with a shorter time until disease recurrence.

The results of our current study suggest that OCT4A expression may be a marker of poor prognosis in bladder cancer. This is consistent with our earlier results of pilot studies conducted on a small number of samples [27] and with papers by other authors [15,19,39,40]. The process of neoplastic malignancy is usually evaluated based on the histological features, including the histological structure, the so-called tissue architecture, of the tumor. In our study, we found that the expression of the OCT4A in cancer cells was related to the tumor tissue architecture, which suggests that the architecture may influence its malignancy. The low percentage of OCT4A-positive cells in tumors at the pT1 stage corresponds to the clinical finding that these tumors usually do not exhibit histological aggressiveness (Figure 2 and Figure 3). By contrast,* in situ* cancers and cancers with an* in situ* component are associated with an unfavorable clinical course [4,5,41,42].

In our study, the percentage of both low- and moderate-intensity OCT4A-positive cells in these cancers was significantly higher (Figure 2), which may be related to their lower histological maturity (Figure 4). This may suggest that the stronger expression of OCT4A as a transcription factor may be an indicator of its stronger regulatory influence on OCT4A-positive cancer cells and, therefore, could strengthen the stem cell phenotype. In our study, we observed that cells with moderate OCT4A expression represented a significantly higher percentage of pTis and pT2 tumors (Figure 2C).

For predicting progression, the clinical evaluation of pTis and pT2 tumors is not straightforward. It is known that a non-invasive* in situ* lesion progressing to the invasive process (first stage of progression) is associated with a less advantageous clinical course to such an extent that preventive cystectomy is justified at the non-invasive lesion stage. The second stage of a higher grade of malignancy of a urothelial tumor (second stage of progression) is the invasion of the muscle layer (muscularis propria) in the bladder wall, the first step of which is pT2 advancement. It is possible that strengthening of the OCT4A expression in pTis (pTis* vs.* pT1, *p* = 0.0154, Figure 2) and pT2 (pT2* vs.* pT3-4 (tumor extent including perivesical fat (pT3) or perivesical organs (pT4)) and metastases, *p* = 0.0082, data not shown) border lesions may be related to completion of the progression to malignancy or may directly precede it and, as such, may be helpful in assessing the individual risk of cancer progression.

The large variability in the percentage of OCT4A-positive cells in individual cases and the lack of significant differences in widely-defined case categories may misleadingly suggest that evaluation of OCT4A expression may not be informative in urothelial bladder cancers (e.g., metastatic *vs.* non-metastatic primary tumors) (Table 1). However, more detailed analysis of the metastatic process (third stage of progression) allows the determination of a tendency towards strengthening of the moderate OCT4A expression in primary tumors involving greater numbers of regional lymph nodes (Figure 1A–C). An increase in the percentage of OCT4A-positive cells in tumors with a lower grade of histological maturity (high grade),* i.e.*, in tumors of greater biological malignancy, may be associated with an inhibiting effect of the OCT4A expression during the process of cancer cell maturation (Figure 4). An increase in the percentage of OCT4A-positive cells in tumors, in which the NDN was >1 (Figure 6), may suggest a stimulating effect of the OCT4A phenotype on the differentiation process of cancer cells. It is possible that along with the increasing percentage of OCT4A-positive cells in successive types of local invasion (Figure 5), the increasing aggressiveness of invasion reflects an increasing migration ability of cancer cells with the stem cell phenotype.

## 4. Materials and Methods

### 4.1. Patients

Ninety patients who had received a cystectomy for urinary bladder cancer at the Oncology Centre, Professor Franciszek Łukaszczyk Memorial Hospital in Bydgoszcz, Poland, in 2005–2010 were enrolled in the study. The patients were 17 women and 73 men aged 44.5–83.8 years (average: 64.6 ± 8.6 years). From each urinary bladder, at least two representative sections of tumor tissue and with adequate urothelium were analyzed. In cases of pN > 0 (*n* = 23), a third section was collected from a metastasis to a regional lymph node. This study was approved by the Committee of Ethics of Scientific Research of Collegium Medicum of Nicolaus Copernicus University, Poland.

### 4.2. Immunohistochemical Staining of Sections

Expression of OCT4A protein was analyzed using the immunohistochemical method described previously [27]. In brief, standard 4–5-µm sections were deparaffinized and hydrated and then subjected to heat-induced epitope retrieval in pH 9 Tris/EDTA (Tris (hydroxymethyl) aminomethane/ethylenediamine tetraacetic acid) buffer. The sections were then incubated with mouse monoclonal anti-OCT4A antibody, a clone recognizing amino acids 1–134 in human OCT4 (Santa Cruz Biotechnology, Inc., Santa Cruz, CA, USA), at a 1:200 dilution in TBS with 1% albumin. Sites of anti-OCT4A antibody binding were visualized with the EnVision system (Dako, Carpinteria, CA, USA) with DAB as the substrate for horseradish peroxidase. After labelling, the sections were stained with hematoxylin, dehydrated and embedded in a relevant medium (Consul Mount; Thermo Fisher Scientific Inc., Waltham, MA, USA). Sections of seminoma tissue were used as a positive control.

### 4.3. Evaluation of Immunohistochemically Stained Sections

**Figure 7:**
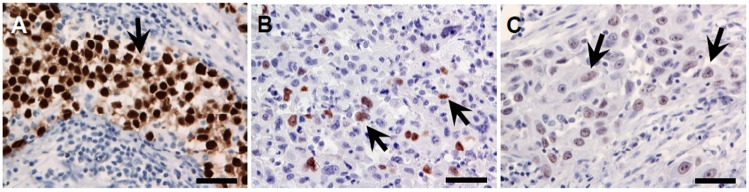
Representative immunostaining with predominant high ((**A**), in seminoma tissue), moderate ((**B**), in bladder cancer tissue) and low ((**C**), in bladder cancer tissue) staining intensity. Arrows show OCT4A-positive nuclei (visualized with DAB (brownish) and counterstained with hematoxylin (blue)); scale bars: 50 µm.

The intensity of nuclear OCT4A expression was evaluated by two researchers (Wojciech Jóźwicki and Anna A. Brożyna) using a previously described scale [27] as follows: 0 = no staining, 1 = low staining intensity, 2 = moderate staining intensity and 3 = high staining intensity (Figure 7A–C). The percentage of cells exhibiting OCT4A expression per 1000 cancer cells was also determined. A high OCT4A expression level was observed only in control seminoma samples.

### 4.4. Tissue Invasion Type

The TIT was classified as described previously [27]. Tissue invasion was evaluated using a scale that ranged from the least to the most aggressive type of invasion, *viz*., frontal, focal, styloid or dispersive (Figure 5B–E).

### 4.5. Statistical Analysis

The relationships between the number of OCT4A-positive nuclei and pT, TIT and NDN were analyzed. Statistical analyses included one-way ANOVA, *t-*test and Pearson’s correlation and were performed using the Prism (version 4.00; GraphPad Software, San Diego, CA, USA) and STATISTICA data-analysis software (version 8.0; StatSoft, Inc., Tulusa, OK, USA). A *p*-value <0.05 was considered significant.

## 5. Conclusions

Our study shows that the phenotype of OCT4A stem cells is strongly related to the histoclinical aspects of urothelial cancer malignancy. The most important morphological aspects of the tumor structure, including its differentiation (NDN), grading and TIT, and successive malignancy progression stages seem to depend on the expression of the stem cell phenotype. In the diagnostic process, information about the percentage of OCT4A-positive tumor cells could facilitate the selection of the treatment mode in cases of borderline pTis–pT1 and pT1–pT2: a higher percentage of OCT4A-positive cells should support more radical therapy. Evaluation of this phenotype may also be useful for determining the prognosis on an individual basis, and control of this phenotype in cancer cells may represent an important objective for new therapeutic technologies. Further studies on human urothelial bladder cancer tissue samples, involving molecular techniques, such as quantitative PCR linked to laser-assisted microdissection and cell-based experiments, especially in primary bladder cancer cells, are needed to clarify the molecular role of OCT4A in the biology and behavior of bladder cancer cells.
